# Computational Analysis of Telomerase RNA Evolution in *Caenorhabditis* Species

**DOI:** 10.3390/ncrna12010006

**Published:** 2026-02-11

**Authors:** Christopher Klapproth, Franziska Reinhardt, Peter F. Stadler, Sven Findeiß

**Affiliations:** 1 ScaDS.AI Leipzig, Humboldtstraße 25, D-04105 Leipzig, Germany; christopher@bioinf.uni-leipzig.de; 2 Bioinformatics Group and Interdisciplinary Center of Bioinformatics, Department of Computer Science, Leipzig University, Härtelstrasse 16-18, D-04107 Leipzig, Germany; franziska@bioinf.uni-leipzig.de (F.R.); peter.stadler@bioinf.uni-leipzig.de (P.F.S.); 3 Max Planck Institute for Mathematics in the Science, Inselstraße 22, D-04103 Leipzig, Germany; 4 Institute for Theoretical Chemistry, University of Vienna, Währingerstraße 17, A-1090 Vienna, Austria; 5 Facultad de Ciencias, Universidad Nacional de Colombia, Bogotá 111321, Colombia; 6 Santa Fe Institute, 1399 Hyde Park Rd., Santa Fe, NM 97501, USA

**Keywords:** telomerase RNA, *Caenorhabditis*, Nematoda, germline specific gene, non-coding RNA, secondary structure

## Abstract

**Background/Objectives:** The telomerase RNA (TR) is an indispensable part of the telomerase protein complex responsible for telomere elongation in most eukaryotic species. Although the telomere terminal repeat sequence (TTAGGC)_n_ in *Caenorhabditis elegans* has been known for years, a telomerase RNA gene was not identified in the entire phylum of Nematoda until recently. **Methods:** In this exploratory study, we employ a combination of different approaches to identify likely telomerase RNA candidates among putative non-coding transcripts. **Results:** A detailed analysis of our prime candidate shows compelling evidence that it encodes the missing RNA element of the telomerase complex, which is notably located in an intron of the coding gene *nmy-2*. Using *nmy-2* homologs in other nematodes as anchors, we annotate the conserved TR gene in 21 *Caenorhabditis* species. We furthermore show that the intronic localization of the TR gene is conserved in two distinct branching groups of the *Caenorhabditis* phylogeny and demonstrate that this property likely emerged from a single point of origin. **Conclusions:** While the intronic TR represents a very interesting evolutionary adaption that seems to have been successful in the Elegans and Japonica groups, the question regarding the macroscopic nematode TR evolution remains.

## 1. Introduction

Composed of an catalytic active Telomerase Reverse Transcriptase (TERT) protein and the template providing telomerase RNA (TR), the telomerase complex represents a common solution to the DNA end-replication problem of linear chromosomes. After each replication cycle, specific telomeric DNA repeats are added to compensate for the continuous shortening process. While the telomerase machinery is typically considered to be highly conserved in Metazoa, in rare instances, such as *Drosophila melanogaster*, alternative telomere maintenance systems have coevolved.

**Figure 1:**
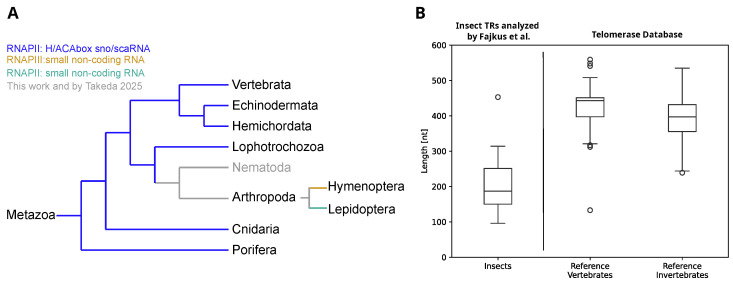
(**A**): Overview of the current understanding of TR evolution in Metazoa (image adapted from [2]). The majority of known TRs in the kingdom of Animalia is of the H/ACA box snoRNA sub-type and transcribed by RNA Polymerase II (RNAP II), indicated in blue. Interestingly, in Arthropoda, and more precisely in the order Hymenoptera, RNA Polymerase III (RNAP III) dependent TRs are reported [1] while in Lepidoptera, RNAP II seems to be responsible for TR transcription [4], indicated in yellow and green, respectively. Corresponding TRs do not contain the H/ACA box feature and are comparably small with respect to other metazoan TRs. (**B**): Length distribution of recently identified TRs in insects [1] (left) compared to known TRs in other Metazoa (right), based on the Telomerase Database [6], last update August 2025.

Recently, the current understanding of divergent telomerase RNA (TR) evolution was summarized by Fajkus et al. [1], as well as Chen and Wellinger [2]. In most Metazoa, TRs are commonly classified as H/ACA box containing RNA Polymerase II (RNAP II) transcripts; see Figure 1A. Besides other common features, TRs of this class have a hairpin structure surrounded by the sequence motifs ANANNA and ACA close to their 3’-ends. These motifs are characteristic features of so-called H/ACA box sno-/scaRNAs [3]. Interestingly, H/ACA box containing TRs utilize the biogenisis pathway of this non-coding RNA (ncRNA) class [2]. Although known TRs of Lophotrochozoa contain the H/ACA box element, TRs recently identified in arthropods lack this feature [1,4]. With a median length of 187 nucleotides [1], at least in Hymenoptera and Lepidoptera, the majority of reported insect TRs are much shorter than those of other Metazoa [5] (Figure 1B). Interestingly, in Lepidoptera, TRs are transcribed by RNAP II [4] while in Hymenoptera TRs are reported to be expressed as RNAP III transcripts [1]. Despite extensive efforts, Nematoda, located between Lophotrophozoa (long H/ACA element containing TRs) and Arthropods (comparably short TRs), represented until recently a blank spot in our current map of animal TR evolution, Figure 1A.

In the nematode model organism *Caenorhabditis elegans* (*C. elegans*), the existence of a transcribed TR was anticipated. The central reason for this is that the TERT protein is well annotated (in *C. elegans*), showing a catalytic *trt-1* domain similar in function to the one identified in those animals using an RNA-protein complex for telomere elongation. However, aside from the catalytic domain, there are structural differences in the rest of the protein in typically conserved amino acids associated with TR binding [7]. Although extensive efforts made to identify TR genes in Metazoa [8], identification of a nematode homolog remained unattainable for a long time. One contributing factor is certainly the substantial age of the Nematoda phylum, which initially diverged over 500 million years ago [9]. A likely explanation would thus be a different overall structure of the TR compared to those in other animals [2]. This possibility has hindered progress in this field of research, as the highly heterogeneous nature of TRs makes it difficult to infer what properties to look for in a given candidate gene.

Due to the large evolutionary distance between sequenced nematodes and the next related class with an abundance of known TR gene, Insecta, a homology-based search for this gene is not viable. We present a computational workflow based on an exhaustive multi-stage filtering process which investigates different features of putative RNA transcripts in the model organism *C. elegans*. After removing known genes and putative novel coding transcripts from our candidate pool, we analyze plausible TR template regions corresponding to the well known (TTAGGC)_n_ telomeric DNA repeat of *C. elegans*. We identify conserved secondary structure elements and furthermore annotate candidate sequences with H/ACA box-like motifs. The small number of final candidates contain the features that one would expect to find in the genuine TR gene. Interestingly, the most promising TR candidate is localized in the intron of the germ-line-upregulated *nmy-2* gene, encoding the Non-muscle Myosin 2 protein in *C. elegans*. Tracing the evolutionary lineage of this intronic TR gene, we discovered homologs in 21 *Caenorhabditis* species of the Japonica and Elegans groups. Remarkably, the intronic TR appears absent in all 12 analyzed previously branching *Caenorhabditis* species. Notably, the sequences of the 21 identified homologs are highly diverse and are not suitable for a Blast search against each other.

Our study is purely computational and the identification of potential TR genes is based on available RNAseq and genomic data. However, the recently published study of Takeda et al. [10] confirmed our prime candidate as a functional TR in *C. elegans*. They investigated the TR gene in great detail and verified TERT binding, splicing dependence, interaction with the dyskerin complex – essential for H/ACA snoRNA biogenesis – and the dependence on germline-expressed genes. Furthermore, they analyzed sequence and structure conservation of three TR homologs, i.e., in *C. elegans*, *C. briggsae* and *C. japonica*. Already, the comparison of these three homologs shows a lot of variation not only at sequence but also at structure level (Takeda et al. [10]; Figures 3 and S3), clearly supporting our results.

In summary, the contributions of Takeda et al. [10] and us represent the first steps towards a comprehensive understanding of telomerase RNA evolution in Nematoda. The presence of intronic TR homologs can be shown in a subset of *Caenorhabditis* species, while the location of the TR gene in other nematodes remains unknown.

## 2. Results

### 2.1. TR Gene Annotation Workflow

**Figure 2:**
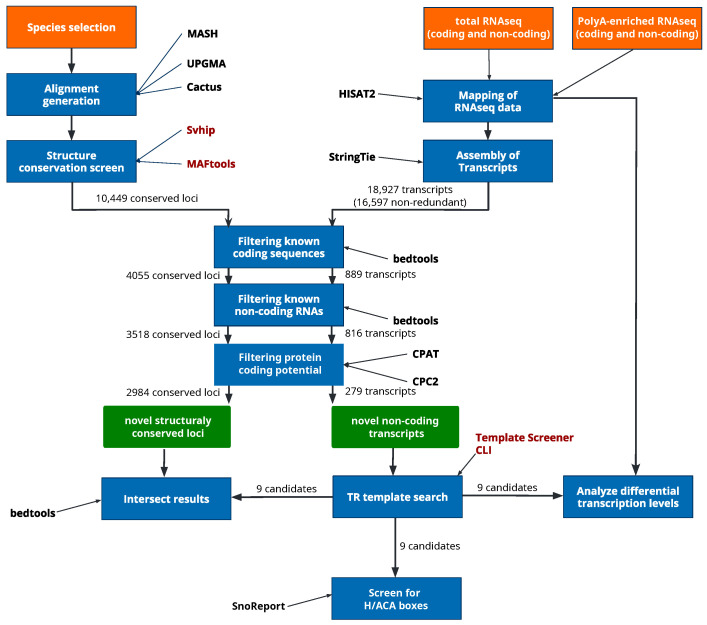
Schematic representation of the applied filtering pipeline. Two independent data sets are initially generated: a screen for conserved RNA structures utilizing Svhip (**left**) and transcriptome analysis (**right**). The comparative genomics approach starts with the process of species selection and whole genome sequence alignment generation utilizing the tools MASH and Cactus. Resulting alignments are post-processed with MAFtools and finally classified with Svhip. After library selection, the RNAseq analysis starts with mapping and subsequent transcript assembly by applying HISAT2 and StringTie, respectively. To identify novel non-coding genes from both sets, i.e., the predicted loci and assembled transcripts, elements that have any overlap with known coding or non-coding genes are removed. Furthermore, CPAT and CPC2 are applied to remove potential unannotated protein coding sequences. Subsequent to this filter process common features of known telomerase RNAs, e.g., length restriction, presence of a valid template region and H/ACA boxes, are evaluated. Presumably the TR is not well conserved on sequence level. However, parts of it might be, and the intersection of both novel transcripts and structured RNA loci could be indicative for such candidates. For the presented study the number of conserved loci and assembled transcripts before, during and after the filter process are indicated along the arrows. In house developed tools are highlighted in red. Svhip is already published, MAFtools is available on github and the TemplateSearcher script is part of the Supplementary Materials. Input is indicated in orange, intermediate filtering steps in blue and the resulting set of novel conserved loci as well as non-coding transcripts in green.

Our approach is based on a multi-stage filtering of potential candidates, investigating different features of RNAs transcribed in *C. elegans*, Figure 2. First, we utilize publicly available RNAseq data collected with different protocols and assemble a candidate transcriptome. We differentiate raw RNAseq data from both Poly(A)-enriched and non-enriched libraries, to distinguish between coding and non-coding types of assembled transcripts. In this regard, we expect that telomerase RNA (TR) can only be found (or show vastly higher coverage) in a library that is not specifically enriched for mRNAs. Second, initial candidate sequences are identified by searching for possible template sites in assembled transcripts using a cyclic permutation of the known (TTAGGC)_n_ telomeric DNA repeat in *C. elegans* [11] as search pattern. Candidates with promising template sites were then further filtered for read coverage, coding potential and possible intersection with known genes. Third, evolutionary signatures of conserved RNA secondary structure elements are evaluated, as it is known that TR shows distinctive structural properties in most annotated species so far: a conserved hairpin structure upstream of the template region, presumably a pseudoknot downstream of it [12,13] and depending on the actual biogenisis pathway of the TR transcript, elements similar to H/ACA or C/D box snoRNAs might define the 3’-end of promising candidates. To identify conserved structural elements on a genome wide scale, we apply the Svhip program [14]. This comparative genomics approach depends on high quality input alignments but is independent of any transcriptome data. Features such as snoRNA-like elements or a conserved hairpin structure upstream of putative template regions are expected to be (at least partially) recovered using this approach. Individual steps are outlined in more detail in the corresponding Section 4. The pipeline described here is modular and individual steps might be skipped, e.g., transcriptome analysis, if sufficient data is unavailable.

For *C. elegans*, as a long-standing model organism, a lot of data is available. In this study, two independent RNAseq data sets published by Hillier et al. [15] and Schreiner et al. [16] have been utilized. If sufficiently transcribed, we expect the putative TR transcripts to be present in the data provided by Schreiner et al. [16], originating from a study investigating remodeling processes of the coding and non-coding *C. elegans* transcriptome under heat shock. Only the control set under normal temperature conditions was used, as TR transcription and telomerase activity should be present under normal living conditions. We compare assembled transcripts of both coding and non-coding RNA, as we would expect TR not to be present in Poly(A)-enriched RNAseq data sets (here: Hillier et al. [15]), thus allowing us to reduce noise and false positives by direct comparison. Although total sequencing depth is substantially higher in the Poly(A)-enriched data set, the number of mapped reads scales proportional to the total amount for almost all chromosomes, Figure S1.

Using StringTie [17], a total of 27,765 putative transcripts was assembled from the non-Poly(A) enriched sequencing libraries. Enforcing a minimum base coverage of ten and filtering for redundancy leaves 18,927 and 16,597 transcripts, respectively. This set contains both coding- and non-coding transcripts. The overall recall rate of 68% (93,593/137,708) for annotated coding sequences (CDS) and 44% for known ncRNA families (Table 1), indicates a reasonable coverage of both classes. Removing all assembled transcripts either overlapping with annotated CDS or ncRNA genes reduces the number of potential TR transcripts to 816. These remaining transcripts potentially represent novel ncRNAs, originating from intronic or intergenic regions with respect to current wormBase annotation. Note that the filtered set might contain reported ncRNA transcripts not belonging to any of the families listed in Table 1. This is on purpose, as remaining ncRNA classes and long non-coding RNAs (lncRNAs) are very diverse and the risk of overlooking a promising TR candidate needs to be minimized. Furthermore, remaining candidates were screened for putative coding sequences and any transcript classified as coding by CPAT and/or CPC2 was removed from the set of transcripts, leaving 279 TR candidates at this stage. Both tools are frequently used to distinguish putative lncRNAs from coding transcripts [18].

As is known, TRs observed in animals are between 200 and 500 nt in length, Figure 1, only sequences of at most 1000 nt were further analyzed. In the next step, candidate transcripts were screened for plausible template regions matching the known telomeric repeat of *C. elegans*. Circular permutations of the *C. elegans* telomere terminal repeat TTAGGC where searched utilizing the in-house TemplateSearcher script (available in the Supplementary Materials). Length cutoff for template regions was set to be between 8 and 20 nt. Given the six nt long DNA terminal repeat, resulting annealing sites are between 2 and 14 nt in length. This way, 13 transcribed template containing TR candidates were identified. Four of these candidates contained multiple template sites, which is rather unexpected for a functional TR gene. The remaining 9 candidates were intersected with the set of structurally conserved loci identified by Svhip, screened for H/ACA box elements utilizing SnoReport2, and their expression was compared to the levels observed in the Poly(A)-enriched sequencing library (Table 2). The results of these analyses were compared to known telomerase RNA genes of other species, in particular with respect to their template position, and genomic context was manually inspected in the genome browser; see Section 4.8.

### 2.2. Genome-Wide Secondary Structure Screen

As a transcriptome independent approach, we analyzed whole-genome sequence alignments applying the Svhip toolkit for conserved secondary structure element prediction,  Klapproth et al. [14]. The fundamental problem with secondary structure conservation screens is the necessity of genomes that are both of high assembly quality as well as evolutionary close enough to still be meaningfully aligned. Therefore, the presented analysis was limited to 20 high-quality genomes of Rhabditida, with *C. elegans* as the reference organism (Table S2). Whole-genome alignments are typically highly fragmented. Therefore, our in-house developed MAFtools (https://github.com/chrisBioInf/MAFtools last update 25 June 2025) was applied to remove gap-rich blocks and merge adjacent alignment blocks by removing individual sequences, thereby creating a consensus set of species per block. While this process slightly reduces the overall number of aligned species per block, it drastically improves cohesiveness of the genome alignment. The output contains continuous blocks of several hundred nt each instead of many more that are only a few nt long (Figure S3).

Resulting alignments were processed and analyzed with Svhip. In total, 10,449 loci showing patterns of structurally conserved RNA elements were predicted. Intersecting these loci with known annotated ncRNA families of *C. elegans* shows an overall recall within expectation (Table 1). The seemingly low recall for snoRNAs here is explainable by the large detection discrepancy between H/ACA box and C/D box snoRNAs, as noted in a different work [14]. Regardless, the observed recall rates are in accordance to a similar screen by Missal et al. [19], which was based on two genomes only.

The same filtering steps as for the StringTie assembled transcripts were applied to remove any Svhip predicted loci that overlap with annotated CDSs, ncRNAs or encode (parts of) potentially unannotated proteins (Figure 2). The remaining 2984 loci likely represent novel structured RNA elements. These are, however, not the main focus of this study, and we only report them for completeness.

### 2.3. Inspection of TR Candidates

The RNA sequences listed in Table 2 present the most likely candidates for the *C. elegans* TR considering many different factors. However, TR_C2 in particular displays all properties expected from the genuine TR gene. Other candidates are likely spurious either because of their template location, having multiple template regions or displaying unlikely transcription patterns in available RNAseq data.

TR_C1 shows very low overall transcription coverage, which is also similar to the one observed in the Poly(A)-enriched transcriptome data, making it an unlikely fit. One would expect that the genuine TR transcript should ideally not be found in a Poly(A)-enriched library. However, a similar indistinct transcription pattern can be observed for TR_C4, TR_C5 and TR_C7, making them unlikely candidates as well. Furthermore, TR_C7 partially overlaps with LITAF domain-containing protein annotated in previous WormBase versions. An extreme case is TR_C3 which is highly abundant in the Poly(A) enriched library and seems to correspond to a much longer transcript of a FBA_2 domain-containing protein. For candidates TR_C6, TR_C8 and TR_C9, the identified template positions are not at the expected close proximity of the transcripts 5’-end. Although TR_C7 and TR_8 have a high-quality putative template-site, their unusual lengths of 927 nt and 988 nt, respectively, make them unlikely TR candidates. Furthermore, observed transcripts are notably present in the Poly(A)-enriched data set. This, in addition to the fact that our primary candidate, TR_C2, showed all other likely properties of a telomerase RNA, lead us to the conclusion that TR_7 and TR_8 are highly likely transcripts of unknown function but not associated with the telomerase machinery.

### 2.4. Properties of the Primary C. elegans TR Candidate

**Figure 3:**
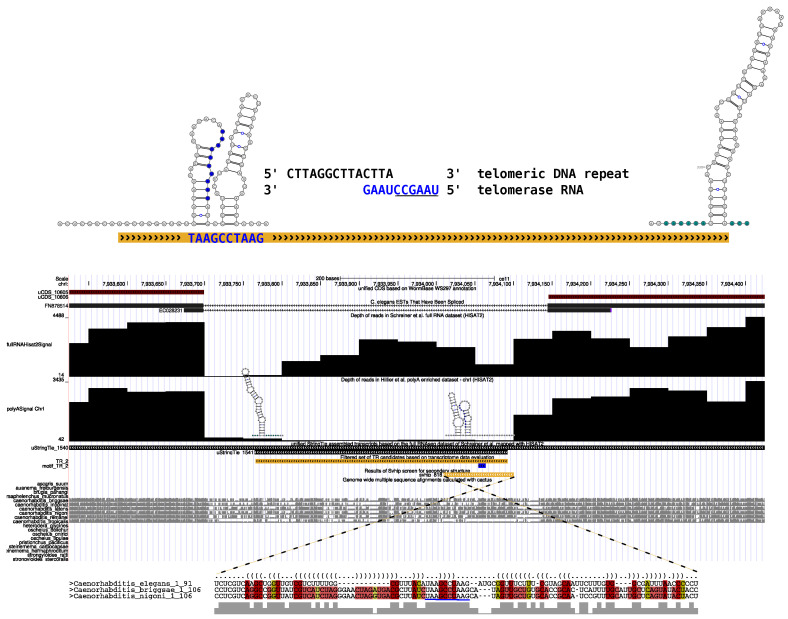
Summary of the TR_C2 candidate located on the reverse strand of chromosome I of *C. elegans*. (**Top**): Schematic representation of TR_C2 depicting two common metazoan TR features: (i) the template region composed of template (underlined) and annealing part and (ii) the predicted H/ACA boxes. Shown secondary structures are predicted on the corresponding sub-sequence of *C. elegans*. Interestingly, the secondary structure at the 5’-end, corresponding to the Svhip locus, does not contain a stem upstream of the template, a feature reported for many other metazoan TRs and also known as template boundary element (TBE) [8]. (**Bottom**): Genomic context of TR_C2 visualized using the UCSC genome browser. All data processed and used in this study is provided as a custom trackhub and partially displayed here. WormBase annotated CDSs are shown in dark red. The second track is part of the available UCSC data and depicts spliced expressed sequence tags (ESTs). Both tracks in combination clearly indicate an intronic origin of TR_C2. In the middle section, we show signals of the two analyzed RNAseq data sets, with Poly(A)-enriched at the top [15] and full RNAseq at the bottom [16]. Assembled transcripts of the latter are also shown. The long transcript (uStringTie_1540) presumably corresponds to the mRNA transcript while the shorter one (uStringTie_1541) is an independent transcript originating from the intron. The corresponding, most promising TR candidate (yellow) including its respective template region (blue) and a conserved secondary structure signal are shown below. Interestingly, the start of the conserved structural element is slightly shifted (chrI: 7,934,010–7,934,100) in comparison to the start of the assembled transcript (start at 7,934,092). The Svhip scored alignment, including conserved base-pairing nucleotides (colored bases) and the resulting consensus secondary structure in dot-bracket notation, is shown below the conservation signals of the original whole genome alignments (gray track).

The candidate TR_C2 is striking in that it, aside from having a high transcription level, unifies multiple additional ’soft’ filtering constraints: First, its length of 327 nt is well within the range of 200 to 500 nucleotides for telomerase RNAs found in many other eukaryotic species. Second, assuming the transcript is completely covered in the full RNAseq library, its notable absence in the Poly(A) enriched sequencing library lends additional support to it being a somewhat independent non-coding transcript. Additionally, the predicted template site is close to the 5’-end. Figure 3 graphically summarizes the observed features of TR_C2, including a secondary structure as predicted by Svhip around the template region and terminal H/ACA boxes at the 3’-end predicted with SnoReport2. It has to be noted that none of the remaining candidates listed in Table 2 shows such a complete set of convincing TR features.

Consider the established TR template CUAACCCUAA of most vertebrates, including human [20]. Animals with this TR template share, to the best of our knowledge, the telomere terminal repeat of TTAGGG. Since *C. elegans* has the established DNA telomeric repeat TTAGGC, one would, in the simplest evolutionary scenario, expect a change of the template sequence to the motif GUAACCCUAA. The sequence UAAGCCUAAG of TR_C2 is, however, equally valid for the motif TTAGGC, given the cyclical permutation structure of terminal DNA repeats. Therefore, TR_C2’s template sequence UAAGCCUAAG is strikingly similar to the one found in many animals. Insects and Hymenoptera in particular are purposefully excluded, as they trend towards an unusually high and currently only partially understood variance in TRs [1,21].

Notably, TR_C2 is located in the intronic region of a gene encoding the non-muscle Myosin 2 protein in *C. elegans*. This rather atypical location might be part of the reason why telomerase RNA was overlooked for such along time. The possibility of being a yet unannotated exon is excluded by comparing TR_C2’s transcription levels with the Poly(A)-enriched RNAseq data set. In this sequencing library, the putative transcript is notably absent, while the surrounding exons show similar coverage in both analyzed RNAseq libraries; see Figure 3.

In parallel to the presented investigation, an independent study of Takeda et al. [10] experimentally verified that our prime candidate, TR_C2, is indeed the telomerase RNA of *C. elegans*. Besides the TR of *C. elegans*, they investigated homologs in *C. briggsae* and *C. japonica*. Already, the comparison of these three homologs shows considerable variation at sequence as well as structural level, further supporting our claim that homology search alone is an insufficient tool for TR gene annotation. In the following, we focus on the evolutionary aspects of telomerase RNA and their host–gene interaction within nematode species.

### 2.5. Annotation of the TR Gene in Other Caenorhabditis Species

After identifying the most likely candidate for the *C. elegans*, TR annotation of the corresponding gene in other Nematoda species is the obvious next step. A standard homology search using BLAST v2.12.0 was attempted, but yielded mostly inconclusive results. This is unsurprising, given the already weak sequence conservation in the genome alignment (Figure 3). Although present in the scored sequence alignment, the sequence of *C. elegans* does not align particularly well to *C. briggsae* and *C. nigoni* (Figure 3). The latter two species support the existence of a conserved hairpin structure upstream of the template region, presumably corresponding to a TBE or stem P1.1; compare Figure 3 and Section 2.7. Notably, the TR of *C. elegans* diverged, and most of the corresponding bases are missing. The observed weak conservation in combination with the known phenomenon of typically fast evolving TR genes are additional reasons why the TR of *C. elegans* is hard to identify with conventional approaches.

In case of the TR_C2 gene, its location within the second intron of the *nmy-2* gene defines its immediate syntenic region, i.e., the adjacent exons, well. Utilizing the tool ExceS-A [22], corresponding protein homologs of *nmy-2* could be identified in 81 nematode species for which high-quality genomic data was available. In particular, the *nmy-2* gene is conserved in all 34 *Caenorhabditis* species we analyzed for this study. We therefore focused our efforts on using the *nmy-2* intron as a clearly defined anchor for finding the TR gene in related species.

Although the number of introns and the overall structure differ in several *nmy-2* genes, 75 *nmy-2* homologs contain an identifiable equivalent of the second intron found in *C. elegans*. Interestingly, the size of the respective introns is extremely variable already within the *Caenorhabditis* species, and the overall structure of the *nmy-2* gene has some notable distinctions (Figure S4). In particular, the earlier branching *Caenorhabditis* species show a notable fragmentation of exon 5 and exon 12 into many smaller exons. Additionally, the same exon 5 is drastically shortened in a number of species in the Elegans and Japonica phylogenetic group and completely absent in *C. drosophilae*, suggesting some flexibility in structure with respect to its function.

**Figure 4:**
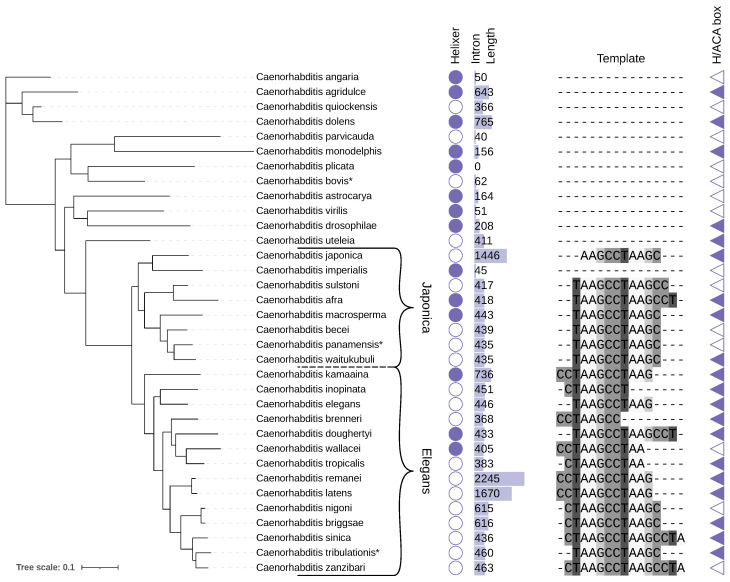
TR evolution in *Caenorhabditis* species. Inferred phylogeny is estimated with IQtree3 [24] and based on 1734 gene orthogroups identified with Proteinortho [25]. Reference protein sequences were taken from the WormBase (v. 297) where applicable. Additionally, a gene annotation was predicted with Helixer (filled circle), coding sequences extracted using bedtools and then translated using biopython. A Helixer annotation likely reduces accuracy compared to curated annotation. The presence of an equivalent of intron 2 in *C. elegans* is indicated by the intron length and a respective bar visualizing this value. The actual number of the intron is different for some species, if for example the first exon of the enclosing protein is merged with the second one and therefore intron 1 is missing. For simplicity, we always refer to it as conserved intron 2 in the text. The existence of a valid TR template within the intron, i.e., a plausibly positioned sequence that is the longest permutation cycle of the reverse complement of TTAGGC, the canonical tandem repeat associated with *Caenorhabditis* telomeres, plus an annealing site of at least length 2, is represented by the respective sequence. The triangle is filled if H/ACA box motifs are predicted by snoReport2. The tree visualization and feature annotation was created with iTol [26]. The asterisk (*) indicates species without an chromosome level assembly.

As pointed out before, intron *length* is highly variable between species, suggesting low evolutionary pressure outside conservation of the TR gene. This is supported by the fact that the intron in the outer phylogenetic group, i.e., the one homologous to *C. elegans*’ intron 2, is on average notably shorter (Figure S5 and Figure 4). In the extreme case of *C. plicata*, the corresponding intron is even completely absent. In multiple other species, the intron would be too short to house the TR at all: examples include *C. angaria* (52 nt), *C. bovis* (64 nt) and *C. virilis* (53 nt). Thus, the rearrangement of the TR gene typically coincided with a substantial lengthening of the intronic sequence. Presumably, the TR was inserted into the already-existing intronic sequence instead of replacing it. This by itself, however, does not explain the comparably enormous length of the intron in some species, i.e., *C. remanei* (2244 nt), *C. latens* (1670 nt) and *C. japonica* (1446 nt), suggesting that the intron sequence is not entirely filled by the TR in these species. This, in turn, calls into question the exact biogenesis pathway of the TR in these species, as splicing of the intron without further post-processing would be presumably insufficient. Following the interpretation by Takeda et al. [10], it is likely that the TR is processed using the RNA exosome-dependent pathway of intronic H/ACA box snoRNAs. However, the majority of the introns in the *nmy-2* gene are notably elongated in these species, which suggests that the presence of the TR is not the only factor here (Figure S4).

Continuing the analysis, the identified sequences equivalent to intron 2 in *C. elegans* were extracted using bedtools and screened for putative TR template regions in the same way as the *C. elegans* candidate transcripts. In total, for 21 of the 75 detected introns (including *C. elegans*), a plausible template sequence for the conserved TTAGGC telomeric DNA repeat was identified (Figure 4). Notably, all of these are closely related *Caenorhabditis* species of the Elegans and Japonica groups, with more distant nematodes not displaying any signs of the TR gene being present in the corresponding intron. For this reason, we limited the following analysis of TR evolution to *Caenorhabditis* species. With respect to groups with and without the intronic TR gene, the one notable exception is *C. imperialis* from the Japonica group, which has no such template as well as a much shorter intron. Another outlier is *C. plicata*, which, as the only *Caenorhabditis* species, has no intron 2-equivalent in its *nmy-2* homolog.

The location of the discovered template region within the intron is very uniform for most species, with the notable exception being the species with overly long introns (Figure S6). The template region is typically located 80-90 nt from the 5’-terminus of the intron, which would be further than in the typical structure found in animal TRs (40–70 nt). This, combined with the existence of the extremely long outliers in *C. japonica* and others, suggests that some form of post-processing of the spliced intron on *both* termini might occur. However, based on the computational evidence at hand, it is not possible to make any definitive statement on this. The distance between template region and downstream intron terminus is typically 300–350 nt. Exceptions are again *C. remanei*, *C. latens* and *C. japonica* (Figure S6).

*Caenorhabditis* phylogeny was estimated independently of the *nmy-2* intron utilizing Proteinortho and IQtree. For species that did not have a viable coding gene annotation available at WormBase (WS297), we inferred a preliminary gene annotation with Helixer. We then annotated the presence or absence of the putative TR template region and H/ACA box motif within the intron for each corresponding species, Figure 4. The inferred phylogeny, in particular the Japonica as well as Elegans groups are congruent with trees found in the literature [23] and, to the best of our knowledge, none of the available reference trees includes all species analyzed here. Strikingly, it appears that the localization of the TR gene in the intron of *nmy-2* is clearly a distinct feature of the Japonica and Elegans groups. TR gene homologs seem completely absent in earlier branching *Caenorhabditis*. Indeed, the tree is remarkably consistent with respect to the presence or absence of the TR in the intron. The only evident counter example here is the already mentioned *C. imperialis*, though it is unclear if this is a failure of the annotation pipeline or the result of some later genome rearrangement event, even though the very short intron size (47 nt) suggests the latter. An issue with the underlying sequencing data cannot be ruled out either.

### 2.6. TR Sequence Features Across Caenorhabditis

The TR template itself, while conserved, shows some notable variation across species (Figure 4). TAAGCCTAAGC is the overall consensus motif, containing TAAGCC as the template site for the GGCTTA/(TTAGGC) telomeric DNA repeat. The highly conserved ’core’ of the template region is, with two exceptions (*C. japonica* and *C. brenneri*), always the sequence TAAGCCT. About half of the species extend the template with the addition of one or two leading C nucleotides, which is particularly common in the Elegans group, where all species except *C. doughertyi*, *C. tribulationis* and *C. elegans* itself share this property. The 5’-end of the template region shows more variability in length, with some instances being very short, i.e., CTAAGCCT (*C. inopinata*) and CCTAAGCC (*C. brenneri*). The longest variation of the template region found here is CTAAGCCTAAGCCTA (shared by *C. sinica* and *C. zanzibari*).

Using snoReport2, we could identify plausible H/ACA-like motifs for most candidates with an intronic template, notable exceptions being *C. sulstoni*, *C. becei*, *C. panamensis*, *C. wallacei*, *C. nigoni*, and *C. zanzibari*. Notably, in some species, we find putative H/ACA box motifs but no viable TR template region, indicating that the intron may house some other non-coding functionality in absence of the TR. Alternatively, snoReport2 is likely not very specific. Except for potentially spurious H/ACA box predictions, homologous introns in the earlier branching *Caenorhabditis* do not show any signatures of TR genes.

### 2.7. Consensus TR Secondary Structure Model

**Figure 5:**
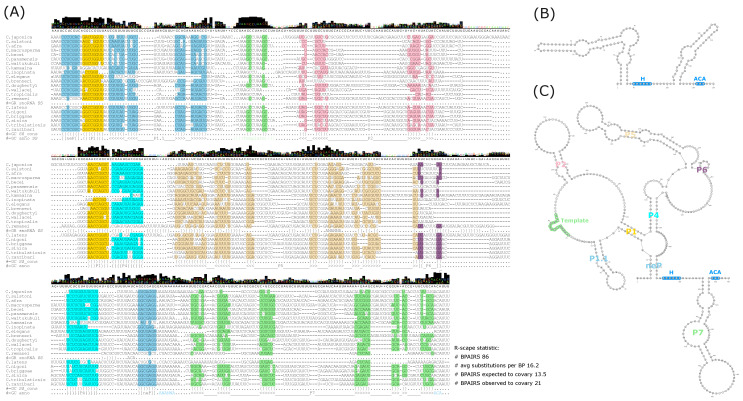
Consensus TR structure model. (**A**) Manually curated template anchored mlocarna alignment. Consensus sequence and nucleotide representatives are shown above each alignment block. Sequences of the alignment are color-coded according to the helices present in the consensus secondary structure, which is indicated below each alignment block (#GC SS_cons). Further, helix annotation according to [10] is indicated in the #GC anno labeled line. Matching parenthesis types correspond to a specific helix and _ positions correspond to unpaired bases of the consensus secondary structure. Additionally, R-scape statistics are listed next to thealignment. Below the sequence of *C. remanei*, the secondary structure of a putative H/ACA box snoRNA is indicated in dot-bracket notation (#GC snoRNA SS). (**B**) The corresponding H/ACA box snoRNA structure predicted with 0.83 confidence by snoReport2. (**C**) Consensus secondary structure representation of the alignment. Annotation of sequence and structure motifs match with the colors and labels of the alignment in (**A**). Sub-figure (**A**) was generated combining the output of the emacs ralee-mode [31] and jalview [32]. VARNA [33] visualizations were generated based on the snoReport2.0 prediction for sub-figure (**B**) and the consensus sequence-structure pair of the TR alignment as input for sub-figure (**C**).

To estimate the gene boundaries of TR homologs in other *Caenorhabditis* species, we used the identified template regions, the Svhip hit, and identified H/ACA boxes as anchor points. We used the sequence 70 nt upstream and 350 nt downstream of each template region as a basis for further analysis. These sequences were aligned for full-length sequence–structure patterns utilizing mlocarna [27]. A hand-curated version (see Section 4.7 for details) of the resulting output alignment is shown in Figure 5.

Based on this alignment, a model for the *Caenorhabditis* TR secondary structure was estimated. In the consensus TR structure, we observe eight distinct stem-loop elements (denoted neP, P1, P1.1, P2 and P4–P7), mostly in line with structural features described by Takeda et al. [10]. According to Rscape [28], covariance is observed for 24% of the identified 86 base-pairs, compared to the expectation of 16% covarying base pairs, Figure 5. A complex secondary structure element is enclosed by the nematode specific stem (neP), linking the 5’-end and the 3’-terminal region immediately upstream of the H/ACA box, compare [10]. This seven-nucleotide-long stem is well conserved across the vast majority of analyzed species. Exceptions are the TRs of *C. kamaaina*, *C. remanei* and *C. latens*. For the latter two species, the 5’-part of the neP perfectly fits the consensus sequence (CCUCGUC) but the 3’-part is missing or at least not aligned. This observation coincides with *C. remanei* and *C. latens* also sharing an abnormaly long TR-hosting intron, allowing for the possibility that the model does not accurately reflect these two species in particular. The P1 stem is well conserved with the exception of *C. kamaaina* and encloses the P1.1 and P2 stems, as well as the mostly unstructured template region. The P1.1 element directly upstream of the template region contains several covarying base-pairs and, with the exception of *C. elegans*, corresponds to a well conserved RNA element. The P4 stem forms an up to 12 base-pair-long helix enclosing P5. In our alignment-based model, P6 is extremely short and most likely unpaired. At the 3’-end of most TRs, the structure element P7, enclosed by H/ACA boxes, is found.

To complement the sequence–structure alignment approach described above, the alignment-free covariance search tool CMfinder [29] was applied to the set of homologous TR sequences. CMfinder aims to identify RNA motifs in a set of unaligned sequences and produces representative covariance models (CMs) for the given input set. Subsequently, cmsearch [30] can be applied in order to identify homologous loci within sequences or complete genomes. In the analyzed TR homologs, CMfinder identified four single helix alignments and corresponding CMs. Only one of these CMs, corresponding to P1.1 and a part of the template sequence in our consensus structure model, was assessed to be representative for the set of identified TR homologs, see Section 4.4.3 for details. Presumably due to the large insert in the loop region of P1.1 in the *C. brenneri* TR, this locus was neither aligned by CMfinder nor recovered by the cmsearch analysis. However, the corresponding sub-sequences of the remaining 20 TRs were recovered and the CMs seems to be representative for the identified homologs. Unfortunately, using the generated CM for genomic screens in earlier branching *Caenorhabditis* species did not reveal any additional TR loci outside the already identified 21 homologs. This suggests that the difference with respect to TRs of earlier branching species is more substantial than initially assumed.

In summary, the presented evidence shows that even within the very limited set of identified *Caenorhabditis* TR homologs, diverse sequences, folding into notably distinct (sub-)structures, evolved. On a smaller scale, this trend is also observed in the structural probing experiments of *C. japonica*, *C. elegans* and *C. briggsae* TR homologs reported by Takeda et al. [10]. With respect to our consensus structure model, TRs of *C. kamaaina*, *C. remanei* and *C. latens* are the most diverged and therefore presumably good targets for further experimental investigations.

## 3. Discussion

We computationally identified the most likely candidate for the until-recently-unidentified TR in *C. elegans*. All analyzed candidates share the required feature of a plausible telomeric DNA repeat template site matching the conserved (TTAGGC)_n_ telomeric DNA tandem repeat. We investigated different properties of putative genes to find the most likely TR candidate, which we identified as TR_C2. Notably, it shares average length, being a high-confidence non-coding sequence, a conserved secondary structure, strong transcription activity and overall positioning of the template site within the transcript comparable to TRs found in many other species. As noted before, the accuracy of our predictions was independently corroborated by Takeda et al. [10].

The most remarkable property of the *C. elegans* TR gene is its location in the intron of a coding gene, something which is highly unusual for this type of ncRNA. While intronic snoRNAs exist in abundance [34,35], this observation is unique in the context of telomerase RNA and raises the question of an evolutionary advantage of this particular adaptation. It is also conceivable that producing the TR as a byproduct of the splicing process of *nmy-2* might counteract a previous mutational loss of an independent promoter. Notably, we could not identify even a degenerate TATA box motif upstream of the putative TR gene in our investigation (see Supplementary Materials). Very intriguing is furthermore the evident conservation of the intronic TR gene in 20 additional *Caenorhabditis* species (21 including *C. elegans*). Interestingly, we observe conservation of the intron in many more distant nematode species. However, the presence of the TR gene indicated by template site and conserved H/ACA boxes seems to be limited to a subset of *Caenorhabditis* species, which are consistent with the emergence of the Elegans and Japonica groups. From this, we conclude that the TR gene was most likely relocated into the *nmy-2* intron during genomic rearrangement shortly before the divergence of these two groups, pointing to a single origin of the intronic TR gene. A reverse scenario, where the intronic localization came about much earlier and was independently *lost* in earlier branching *Caenorhabditis* species is, of course, theoretically also possible, but less likely following a maximum parsimony interpretation. Interestingly, the location of a TR gene in other *Caenorhabditis* species cannot be identified via sequence homology search even within the annotated TRs of the 21 species. The same holds true for other nematodes in our data set. A covariance model based approach is also not able to overcome this limitation. We therefore conclude that the TR homologs could *only* be found because the coding genes conserved intron could be used as a search anchor. Furthermore, a ‘brute force’ approach, as utilized for the initial annotation in *C. elegans*, would not work here, as it inherently relies on the availability of large amounts of high-quality sequencing data and a very reliable reference annotation to filter down candidates. Therefore, a first step in starting this process would come down to generating high-quality annotations for one or multiple of the yet missing species using a pipeline like AUGUSTUS [36] or Helixer as well as sufficient amounts of sequencing data.

While the evidence for the single point of origin for the intronic TR is in our opinion very strong, we point out that the presented Proteinortho inferred phylogeny has to be considered imperfect. It recovers the accepted phylogeny (where it is known) decently well; compare Stevens et al. [23]. Nonetheless, one would assume some isolated species to be placed differently. In particular, we expected *C. monodelphis* to branch earlier than it is the case here. However, the exact phylogeny of the plentiful *Caenorhabditis* species is actually not entirely resolved at the time of writing [23,37], and, to the best of our knowledge, none of the available reference trees includes all analyzed species. The biggest problem for deeper evolutionary analysis of *Caenorhabditis* TRs—and, by extension, also the *nmy-2* gene—is the lack of reliable reference annotation data for many of these species. Note that the Elegans group and the Japonica group are phylogenetically resolved with great accuracy compared to the earlier branching species, which is particularly notable as the density of reference annotations is comparably higher in these groups. Consequently, we had to predict a gene model using Helixer for fewer species in these groups, suggesting that its usage does indeed correlate with a loss in phylogenetic reconstruction accuracy. From this, we conclude that the quality of our tree could be further improved by creating a well curated annotation for the investigated *Caenorhabditis* species. This, however, is unfortunately a very time-consuming task and beyond the scope of this study. Nonetheless, it will be considered for future work.

An additional challenge with respect to a comprehensive evolutionary history of the TR genes in nematodes *generally* are the predicted time scales. According to Qing et al. [9], Nematoda are one of the oldest still existing phyla (initially diverging over 500 million years ago), exacerbating the issue of similarity-based gene annotation. The *Caenorhabditis* genus is currently estimated to have emerged 74 million years ago. Despite their superficial morphological similarity, recent *Caenorhabditis* species in many cases also diverged a relatively long time ago, with estimates for the last common ancestor of *C. elegans* and *C. briggsae* ranging up to over 50 million years ago [38]. The distance between these two species is therefore within the same order of magnitude as the last common ancestor of many recent mammals, e.g., human and mouse (90 million years ago). Notably, *C. elegans* and *C. briggsae* are not even close to being the most distant species in this analysis.

This again aligns well with the difficulty of annotating the fast evolving TRs based on homology searches, as was pointed out before. Interestingly, we note that the *C. elegans* TR, when compared to other TR genes identified by us here, is actually not even a particularly good model for either the consensus sequence or structure (Figure 5). Compared to most other aligned species, *C. elegans* displays a relatively large number of divergent positions, although it is not the most distinct TR (*C. remanei*). Nonetheless, the structure described here, which is supported by base pair covariance, is macroscopically consistent with the SHAPE-derived structure reported by Takeda et al. [10]. We suggest that important secondary structure features are conserved across *Caenorhabditis* species with intron-located TRs. The consensus structure displays certain features that match those observed in other animals, with the P1.1 pre-template stem being a notable example, which is also observed in non-vertebrate metazoans, fungi and plants [8]. Interestingly, the P1.1. stem in particular is notably absent in *C. elegans*, further marking it as an outlier within *Caenorhabditis* TRs. We note that all species analyzed here share the feature of H/ACA boxes, which is in line with other known metazoan TRs.

An open question is the role of the three unusually long template-carrying *nmy-2* introns observed in *C. japonica*, *C. remanei* and *C. latens*. With respect to their atypical length, it is very likely that major parts of both the 3’- and 5’-terminus may become clipped during the TR maturation process. Alternatively, the TR in at least *C. remanei* and *C. latens* would itself be atypically long. The same is, to a lesser extent, true for *C. kamaaina*, where the distance between 3’-terminus of the intron and template region is also atypically large. In general, we follow the interpretation of Takeda et al. [10], that the biogenesis pathway of intronic TRs in *Caenorhabditis* species likely follows that of intronic snoRNAs found in other animals, i.e., there is a post-splicing processing step involving nuclear RNA exosome-dependent digestion at the 3’-end of the intron. Some form of clipping on this terminus is likely to take place in all intonic TRs annotated here, as there is a notable offset between the (conserved) H/ACA box motif and the 3’-terminus of the intron, suggesting leftover space beyond the functional transcript.

As an additional anomaly, the unusually long introns are highly dissimilar with respect to their template localization (Figure S6). The distance between the 5’-terminus of the intron and the template in *C. latens* is 500 nt shorter than this offset in *C. remanei*, for instance. Despite being macroscopically similar in size, the template in the corresponding *C. japonica* intron is offset much further away from the 3’-end and significantly closer to the 5’-end. These divergences make a computational determination of exact TR boundaries very challenging. Additionally, these TRs also diverge notably from the consensus structure (Figure 5), raising the possibility of differences in biogenesis and function.

Covariance models were used to screen more distant nematode species for TR homologs, though without success. With that, the annotation of additional nematode TRs is not straightforward, as neither sequence homology nor covariance models proved sufficient with the data presented here, further illustrating the trend towards rapid diversification of TRs. It thus stands to reason that the best approach for expanding the atlas of nematode TRs would be an adapted exhaustive filtering approach in selected species. This work can thus be seen as an initial proof of concept for such an approach in the Nematoda phylum. A further improvement would potentially be a targeted search for putative template regions in introns of germline genes, if the intron-localization of TRs is a trait shared by other species in this phylum. While interesting, this next step is beyond the scope of this investigation.

## 4. Materials and Methods

### 4.1. Caenorhabditis elegans Transcriptome Assembly

Raw sequencing data of the studies of Hillier et al. [15] and Schreiner et al. [16] was downloaded using fasterq-dump of the sra-tools software package v3.2.1 [39] and the listed accessions; see Table S1. Quality control was carried out using FASTQC and MULTIQC while adapters were removed applying fastq-mcf. Preprocessed reads were then mapped to the *C. elegans* genome using HISAT2 [40]. Expected transcription levels for a number of known genes was confirmed manually utilizing the UCSC genome browser and a custom made trackhub, see Section 4.8. Transcripts were assembled using StringTie [17] with standard parameters and no reference annotation, to focus on putative novel transcripts.

### 4.2. Transcript Filtering

Annotation for *C. elegans* (GCF_000002985.6) was downloaded from NCBI’s FTP server (https://ftp.ncbi.nlm.nih.gov/genomes/refseq/invertebrate/Caenorhabditis_elegans/latest_assembly_versions/GCF_000002985.6_WBcel235/GCF_000002985.6_WBcel235_genomic.gtf.gz). Chromosome encoded ncRNA elements of gene_biotypes snoRNA (346), miRNA (260), tRNA (612), snRNA (129), rRNA (20) where extracted from the gtf file, converted into 6-field (big)bed format and integrated into our UCSC trackhub; see Section 4.8. In total 204,448 coding sequence elements listed as gbkey “CDS” encoded on the six chromosomes where processed in the same way. However, for one genomic region, i.e., identical start, end and strand, multiple CDS elements might be annotated. For the intersection with other elements, e.g., Svhip predictions and assembled transcripts, the set of annotated CDS was reduced to unique loci, leaving 137,708 CDS elements. The corresponding (big)bed files are also integrated into the UCSC trackhub.

Assembled transcripts were further filtered for putative coding regions with CPAT [41] and CPC2 [42]. For lack of a dedicated invertebrate or nematode regression model, we used the pre-built *Drosophilid* model for CPAT. However, testing the other available models yielded only very marginal differences in results, suggesting the overall predictions to be relatively stable. CPC2 was run with standard parameters. Only transcripts that were not classified as non-coding (cutoff: ≤0.5) by either tool were considered for further analysis.

### 4.3. Construction of Rhabditida Full Genome Alignment

Rhabditida species with a chromosome-level assembly that were marked as reference genome in the NCBI were downloaded in 11/2023. If multiple reference assemblies existed, the most recent one was preferred, while also picking pooled or hermaphrodite genomes if sex specific data was available, as is the case with several nematode species. This, however, should not make a large difference, as TR is expected to be transcribed in all sexes. The 20 selected species and corresponding assemblies are summarized in Table S2.

The cactus progressive genome aligner [43] was applied after estimating an initial phylogenetic tree and genome similarity using the program MASH (word size 18, 10,000 samples) [44], see Figure S2. The genome alignment with *C. elegans* as reference species was extracted in multiple alignment format (MAF) using the hal2maf tool of the cactus framework.

The generated MAF alignment was post-processed using MAFtools (https://github.com/chrisBioInf/MAFtools last update 25 January 2025): First, alignment blocks generated by cactus containing mostly gaps were completely removed using the ungap subprogram (maximal gap threshold: 0.9 of all sequences in the block). Second, overall fragmentation of the genome alignment was reduced with the merge program. It merges adjacent alignment blocks if they share the majority of their species (threshold here: minimum 75% species consensus) and genomic locations of aligned sequences are in close proximity (maximum distance between two coordinates: 5 nts). Sequences only present in one of the original blocks are removed during this step to create a consensus in the merged one. While this slightly reduces the overall number of aligned species per block, it drastically improves cohesiveness of the whole genome alignment, by having continuous blocks of several hundred nucleotides each instead of many more that are only a few nucleotides long (Figure S3). The resulting MAF file was converted into a (big)bed format and integrated into the UCSC trackhub.

### 4.4. Secondary Structure Element Detection

#### 4.4.1. Svhip

Genome alignments were processed into overlapping windows (slide length: 40, window length: 120) and each window was screened for secondary structure conservation signals on both strands utilizing the Svhip framework and the basic model [14]. Overlapping hits were clustered, leading to a total of 10,449 predicted loci. Prediction quality was assessed by intersecting these loci with WormBase [45,46] ncRNA annotation of *C. elegans* (Table 1).

#### 4.4.2. snoReport2

snoReport2 [3] was used to predict putative H/ACA box-like termini on candidates. The included H/ACA prediction model with standard parameters was used. For transcripts containing multiple H/ACA-like motifs, the one furthest towards the end of the transcript was chosen for annotation, as snoReport2 is not inherently biased towards any positioning within candidate sequences. Except for a significant H/ACA box snoRNA prediction (*p*-value 0.83) within the *C. remanei* intron, snoReport2 was utilized to mainly predict the two sequence motifs (H and ACA boxes), but not necessarily the secondary structural elements present in H/ACA box snoRNA genes. Screening for C/D box snoRNA motifs, with the corresponding model, did not produce convincing hits on the intronic sequences.

#### 4.4.3. Covariance Model Based Approach

CMfinder version 0.4.1.18 [29] was applied with standard parameters to identify RNA motifs within the unaligned set of TR homologs. Although CMfinder generates up to five single helix alignments by default, only four are found for the TR homologs. These alignments were converted into covariance models (CMs) and calibrated using cmbuild and cmcalibrate with standard parameters, respectively. Both tools are part of the Infernal software suite version 1.1.5 [30]. To validate the applicability of the resulting CMs for TR homolog identification, the Infernal tool cmsearch was used to screen the set of 21 TR sequences and the respective complete genomes. Only if all local as well as global screens recovered the respective part of the TR homologs as a significant hit, i.e., above the inclusion threshold, was the CM deemed to be representative of the given structure. The respective CMfinder alignments, build CMs and cmsearch results are compiled and summarized in the respective sub-directory of the Supplementary Materials.

### 4.5. Homology-Based Annotation in Other Caenorhabditis Species

The amino acid sequence of the annotated Non-muscle Myosin 2 protein (ACC: NP_492186.3) of *C. elegans* was obtained from the NCBI database. ExceS-A [22], a splice-site aware split aligner (https://github.com/frarei312/ExceS-A-An-Exon-Centric-Split-Aligner, last update 04 January 2022)., was applied to annotate the *nmy-2* gene in all sequenced nematode reference genomes downloaded from the NCBI database and in the genome from *C.tribulationis* (Table S3). First, the amino acid sequence of the *C. elegans* protein was searched against its own genome in order to calibrate the ExceS-A parameters. Subsequently all remaining genomes were screened with standard parameters and −16 for the minimal ExceS-A score of splice sites.

### 4.6. Phylogenetic Tree Construction of Caenorhabditis Species

The proteomes for all *Caenorhabditis* species for which a reference proteome was available in WormBase [45] were downloaded. This was the case for 20 species, not all of which had the conserved TR. For the remaining *Caenorhabditis* species in the data set, an initial gene annotation was generated with Helixer [47] using the invertebrate model. Based on the predicted genes, putative coding sequences were extracted, concatenated and translated into amino acid sequences with biopython [48]. The species *C. auriculariae* was excluded from this analysis. While it does have a conserved intron 2 equivalent with no TR template, there is neither a reference proteome nor a chromosome-level genome assembly available. As Helixer becomes less reliable for inputs consisting of shorter contigs, it was not further considered for phylogeny reconstruction. The resulting proteomes of 34 *Caenorhabditis* species (20 with WormBase annotation and 14 annotated with Helixer) were used as input for Proteinortho [25,49]. This tool was applied with standard parameters to identify coding gene orthogroups. In total, 57,949 gene orthogroups were predicted, of which 1734 are represented in all candidate species for this analysis. Alignments were built for all orthologous genes that are represented in all analyzed 34 *Caenorhabditis* species using Clustal Omega v1.2.4 [50]. Finally, a maximum likelihood tree was constructed with IQtree3 [24,51] using the NQ.pfam [52] substitution model.

### 4.7. Multiple Sequence Alignment Generation

Putative TR orthologous sequences have been generated using the identified template region of each intron as an anchor point. Each candidate consists of the template sequence and 70 nt up- as well as 350 nt downstream of it. Based on the extracted sequences, a multiple sequence alignment was generated utilizing mlocarna part of LocARNA 2.0.1 [27].

The original mlocarna alignment was manually curated (utilizing emacs ralee-mode version 0.8 [31]) in order to generate the alignment shown in Figure 5. The mlocarna Stockholm formated output alignment was truncated at the 5’- and 3’-end according to the length of the *C. elegans*, *C. briggsae* and *C. japonica* homologs published in [10]. Further, the sequence order was adjusted according to the estimated phylogenetic tree and template sequences were shifted to align the corresponding positions (Figure 4). The consensus secondary structure element annotation according to Takeda et al. [10] was added for reference (#=GC anno SS). Finally, the secondary structure and the identified H/ACA boxes of a significant snoReport2.0 prediction within the *C.remanei* TR homolog was added and labeled with #=GR snoRNA SS.

### 4.8. Data Visualization

To visualize and share our data, e.g., mapped sequencing experiments, Svhip predictions and TR candidates, within the genomic context, we provide a so called UCSC trackhub. Please go to https://genome-euro.ucsc.edu/cgi-bin/hgHubConnect and change from the “Public Hubs” to the “Connected Hubs” tab. Copy the link https://telomerase.bioinf.uni-leipzig.de/TelomeraseRNACeleHub/hub.txt into the “URL” text field and click “Add Hub”. You will be directly forwarded to the UCSC Genome Browser loading the *C. elegans* assembly. Please add chrI:7933565-7934292 to the “Position/Search Term” text field and click “GO”. The genomic location of TR_C2 will be shown, similar to Figure 3. Both links were accessed on 25 January 2026.

## 5. Conclusions

The work presented here serves to illustrate the highly heterogeneous nature of the TR gene even within closely related species. *Caenorhabditis* species show the rapid mutation and emergence of both sequence and secondary structure elements in a relatively short evolutionary time frame, further serving as an example on why the homology-based search for the elusive TR has often encountered challenges. While the intronic TR represents a very interesting evolutionary adaption that seems to have been successful in the Elegans and Japonica groups, the question regarding the macroscopic nematode TR evolution remains.

## Figures and Tables

**Table 1:** Recall of annotated ncRNA genes. One set represents annotated ncRNAs that significantly overlap with StringTie assembled transcripts. A minimum overlap of 90% was required to count an annotated RNA as recalled. The second set represents the intersection of annotated ncRNA families and loci of secondary structure conservation predicted by Svhip [14]. “Aligned” refers to the corresponding gene being represented in the multiple genome alignment with at least 50% of its length, i.e., being reasonably detectable. A minimum overlap of 50% was required to count an alignment contained RNA as recalled. Note that the recall rates are estimated by enforcing a certain minimum overlap between items while the restrictive filter pipeline removes all items that have any overlap. Therefore, respective counts are not comparable.

Annotation		StringTie		Svhip		
RNA Family	# Genes	Recovered	Recall [%]	Aligned	Recovered	Recall [%]
tRNA	612	216	35%	255	168	67%
miRNA	260	86	33%	113	81	72%
snoRNA	346	229	66%	195	57	29%
rRNA	20	15	75%	4	4	100%
snRNA	129	56	43%	29	20	69%
Total	1367	602	44%	596	330	55%

**Table 2:** Features of nine template containing TR candidates. For each candidate, the genomic location (sorted by chromosome, start, end and strand), the resulting length, the observed template sequence, and their respective start position relative to the 5’-end of the TR candidate is listed. Furthermore, expression in the two RNAseq libraries is summarized as follows: (i) coverage, defined here as the sum of per base read depths in the total RNAseq library, and (ii) in comparison the transcription level in the Poly(A)-enriched sequencing library is indicated as absent, abundant, similar and half. Additionally, the presence or absence of a structurally conserved sub-region and predicted snoRNA elements are indicated by ✓and ✗, respectively. The most promising and further investigated candidate is highlighted in bold.

	Genomic Location					Template		Expression		Prediction	
ID	Chrom	Start	End	Strand	Length	Motif	Pos.	Cov.	Poly(A)	Svhip	H/ACA
TR_C1	chrI	2,700,026	2,700,251	+	226	AAGCCTAAGCC	127	10	similar	✗	✓
**TR_C2**	**chrI**	**7,933,766**	**7,934,092**	**–**	**327**	**TAAGCCTAAG**	**29**	**818**	**absent**	**✓**	**✓**
TR_C3	chrI	12,084,754	12,085,008	–	255	CCTAAGCC	185	10	abundant	✗	✗
TR_C4	chrII	7,392,341	7,392,646	+	306	AGCCTAAGC	288	11	similar	✗	✗
TR_C5	chrIII	698,155	698,470	+	316	AGCCTAAG	84	13	similar	✗	✓
TR_C6	chrIII	11,609,100	11,609,499	–	400	CTAAGCCT	289	55	absent	✗	✓
TR_C7	chrIII	12,868,600	12,869,526	–	927	CTAAGCCT	541	13	similar	✗	✗
TR_C8	chrV	11,606,871	11,607,858	+	988	GCCTAAGC	923	23	half	✗	✓
TR_C9	chrX	16,432,746	16,433,073	–	328	CTAAGCCTAA	269	51	half	✗	✗

## Data Availability

The original contributions presented in this study are included in the article/Supplementary Materials. Further inquiries can be directed to the corresponding author.

## Supplementary Materials

The following supporting information can be downloaded at: https://www.mdpi.com/article/10.3390/ncrna12010006/s1, a PDF file containing all referenced supplemental Tables and Figures (Supplemental_ncrna-12-00006.pdf) and an archive containing additional data and custom scripts (Supplemental_ncrna-12-00006.zip).

## Abbreviations

*C. elegans*	*Caenorhabditis elegans*
*D. melanogaster*	*Drosophila melanogaster*
TR	telomerase RNA
TERT	Telomerase Reverse Transcriptase
MAF	multiple alignment format
ncRNA	non-coding RNA
lncRNA	long non-coding RNA
CDS	coding sequence
nt	nucleotide
RNAP II	RNA Polymerase II
RNAP III	RNA Polymerase III
EST	expressed sequence tag
TBE	template boundary element
CM	covariance model
