# Supplementary material for: Computational Analysis of Telomerase RNA Evolution in Caenorhabditis Species
Source: Noncoding RNA. 2026 Feb 11;12(1):6. doi: 10.3390/ncrna12010006 (PMC12921731; doi:10.3390/ncrna12010006)
Supplement: Supplementary file 1 [file ncrna-12-00006-s001.zip › Supplemental_ncrna-12-00006.pdf]

non-coding RNA

MDPI

Article

Supplemental Information for: Computational analysis of telomerase RNA evolution in *Caenorhabditis* species.

Christopher Klapproth [1–2], Franziska Reinhardt [2], Peter F. Stadler[2–6] and Sven Findeiß [2*]

[1] ScaDS.AI Leipzig (Center for Scalable Data Analytics and Artificial Intelligence), Humboldtstraße 25, D-04105 Leipzig, Germany
[2] Leipzig University, Bioinformatics Group, Department of Computer Science and Interdisciplinary Center of Bioinformatics, Härtelstrasse 16-18, D-04107 Leipzig, Germany
[3] Max Planck Institute for Mathematics in the Science, Inselstraße 22, D-04103 Leipzig, Germany
[4] University of Vienna, Institute for Theoretical Chemistry, Währingerstraße 17, A-1090 Vienna, Austria
[5] Universidad Nacional de Colombia, Facultad de Ciencias, Bogotá, D.C., Colombia
[6] Santa Fe Institute, 1399 Hyde Park Rd., Santa Fe NM 97501, USA
* Correspondence: sven@bioinf.uni-leipzig.de; Tel.: +49-341-9716704

S1. Supplemental Tables

Table S1. List of RNAseq data sets used in this study, notable protocol features, SRA accessions and publication reference. Corresponding UCSC tracks of the mapped data denoted as "Poly(A)-enriched" and "Full RNA" are available in our custom UCSC trackhub, see section 4.8.

Data set	Accessions	Publication
Poly(A)-enriched	SRR006511 - SRR006520	Hillier et al. [1]
	SRR016679 - SRR016683	
	SRR023579 - SRR023581	
	SRR089405 - SRR089407	
	SRR089821 - SRR089823	
	SRR1056307 - SRR1056314	
	SRR125337 - SRR125339	
	SRR125481, SRR125482	
	SRR5024015, SRR5024016	
Full RNA	GSM3893634 - GSM3893636	Schreiner et al. [2]
	GSM3895512, GSM3895513	

Received: 1 December 2025
Revised: 22 January 2026
Accepted: 26 January 2026
Published: 11 February 2026

Copyright: © 2026 by the authors. Submitted to *Non-Coding RNA* for possible open access publication under the terms and conditions of the Creative Commons Attri- bution (CC BY) license (https://creativecommons.org/licenses/by/4.0/).

Table S2. Nematode species selected for multiple sequence genome alignments. For each species the respective GenBank ID and the year of publication is listed. If known the sequenced sex is indicated as well. Resulting alignments have been subsequently analyzed with `Svhip`.

Number	Species	Sex	GenBank Accession	Year
1	*Ascaris suum*	Pooled	GCA_013433145.1	2020
2	*Auanema freiburgensis*	?	GCA_030370435.1	2023
3	*Brugia pahangi*	Pooled	GCA_012070555.1	2020
4	*Bursaphelenchus mucronatus*	?	GCA_025436335.1	2022
5	*Caenorhabditis briggsae*	?	GCA_000004555.3	2014
6	*Caenorhabditis elegans*	Pooled	GCA_000002985.3	2013
7	*Caenorhabditis inopinata*	?	GCA_003052745.1	2017
8	*Caenorhabditis latens*	?	GCA_002259235.3	2023
9	*Caenorhabditis nigoni*	Hermaphrodite	GCA_027920645.1	2023
10	*Caenorhabditis remanei*	Pooled	GCA_010183535.1	2020
11	*Caenorhabditis tropicalis*	Hermaphrodite	GCA_016735795.1	2021
12	*Heterodera glycines*	?	GCA_004148225.2	2021
13	*Oscheius dolichura*	?	GCA_932521035.1	2022
14	*Oscheius onirici*	?	GCA_932521025.1	2022
15	*Oscheius tipulae*	?	GCA_013425905.1	2020
16	*Pristionchus pacificus*	?	GCA_000180635.4	2020
17	*Steinernema carpocapsae*	Pooled	GCA_000757645.3	2019
18	*Steinernema hermaphroditum*	?	GCA_030435675.1	2023
19	*Strongyloides ratti*	?	GCA_001040885.1	2014
20	*Strongyloides stercoralis*	?	GCA_029582065.1	2023

Table S3. Analyzed genomes of the *Caenorhabditis* genus.

Number	Species	Tax ID	Accession	Release Date
1	*Caenorhabditis latens*	1503980	GCA_002259235.3	2023-04-10
2	*Caenorhabditis inopinata*	1978547	GCA_003052745.1	2017-06-22
3	*Caenorhabditis nigoni*	1611254	GCA_027920645.1	2023-01-19
4	*Caenorhabditis tropicalis*	1561998	GCA_043792875.1	2024-10-28
5	*Caenorhabditis becei*	2301260	GCA_050948105.1	2025-06-17
6	*Caenorhabditis panamensis*	2301259	GCA_900883565.2	2019-04-11
7	*Caenorhabditis bovis*	2654633	GCA_902829315.1	2020-04-17
8	*Caenorhabditis auriculariae*	2777116	GCA_904845305.1	2022-02-18
9	*Caenorhabditis afra*	1094335	GCA_963570955.1	2023-10-27
10	*Caenorhabditis imperialis*	1094324	GCA_963572205.1	2023-10-27
11	*Caenorhabditis japonica*	281687	GCA_963572235.1	2023-10-29
12	*Caenorhabditis doughertyi*	1094321	GCA_963572265.1	2023-10-29
13	*Caenorhabditis drosophilae*	96641	GCA_963572285.1	2023-10-27
14	*Caenorhabditis uteleia*	2305860	GCA_963573275.1	2023-10-29
15	*Caenorhabditis plicata*	281681	GCA_963931815.1	2024-02-06
16	*Caenorhabditis wallacei*	1094326	GCA_963932035.1	2024-02-07
17	*Caenorhabditis sinica*	1550068	GCA_963932045.1	2024-02-07
18	*Caenorhabditis macrosperma*	1094328	GCA_963932285.1	2024-02-07
19	*Caenorhabditis sulstoni*	2305862	GCA_963966605.1	2024-02-24
20	*Caenorhabditis zanzibari*	2306312	GCA_963966625.1	2024-02-24
21	*Caenorhabditis parvicauda*	2305859	GCA_963978915.1	2024-03-10
22	*Caenorhabditis brenneri*	135651	GCA_964036135.1	2024-06-06
23	*Caenorhabditis virilis*	1094323	GCA_964036255.1	2024-05-12
24	*Caenorhabditis monodelphis*	1094320	GCA_964197825.1	2024-07-08
25	*Caenorhabditis quiockensis*	2305861	GCA_964198105.1	2024-07-12
26	*Caenorhabditis kamaaina*	1094325	GCA_964211945.1	2024-11-08
27	*Caenorhabditis angaria*	860376	GCA_964213925.1	2024-08-22
28	*Caenorhabditis agridulce*	3233994	GCA_965217555.1	2025-03-23
29	*Caenorhabditis waitukubuli*	2305864	GCA_965219145.1	2025-03-23
30	*Caenorhabditis dolens*	2035512	GCA_965231745.1	2025-03-29
31	*Caenorhabditis astrocarya*	2035511	GCA_965282815.1	2025-05-18
32	*Caenorhabditis elegans*	6239	GCF_000002985.6	2013-02-07
33	*Caenorhabditis briggsae*	6238	GCF_000004555.2	2014-07-02
34	*Caenorhabditis remanei*	31234	GCF_010183535.1	2020-02-10
35	*Caenorhabditis sp. 40 LS-2015*	1737337	GCA_900536305.3	2019-04-11

S2. Supplemental Figures

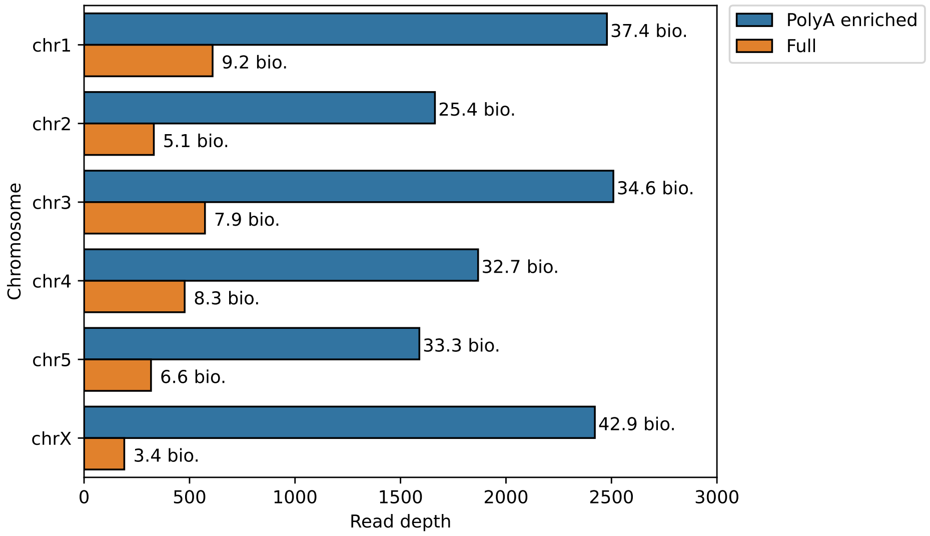

Figure S1. Mapping statistic for two RNAseq data sets, i.e. a `Poly(A)-enriched` library published by Hillier et al. [1] and an untreated `Full` transcriptome published by Schreiner et al. [2]. Read depth of mapped RNAseq data obtained by dividing the number of mapped reads per nucleotide by the chromosome length. The total number of Poly(A)-enriched mapped reads is substantially higher, as the number of sequenced reads in the Poly(A) data set also is. For comparison, we added the total number of billions of mapped nucleotides for each chromosome to the tip of each bar. Except for chromosome X, the total amount still scales proportionally across chromosomes. The Poly(A) enriched data set contained 788 million reads (after quality control), of which 87.4% were mapped to the genome. The data set including non-coding RNAs had 383 million reads, of which 77.2% were mapped.

S3. Abbreviations

C. elegans *Caenorhabditis elegans*
TR telomerase RNA
ncRNA non-coding RNA
nt nucleotide

References

1. Hillier, L.W.; Reinke, V.; Green, P.; Hirst, M.; Marra, M.A.; Waterston, R.H. Massively parallel sequencing of the polyadenylated transcriptome of *C. elegans*. *Genome research* **2009**, *19*, 657–666. https://doi.org/10.1101/gr.088112.108.
2. Schreiner, W.P.; Pagliuso, D.C.; Garrigues, J.M.; Chen, J.S.; Aalto, A.P.; Pasquinelli, A.E. Remodeling of the *Caenorhabditis elegans* non-coding RNA transcriptome by heat shock. *Nucleic acids research* **2019**, *47*, 9829–9841. https://doi.org/10.1093/nar/gkz693.

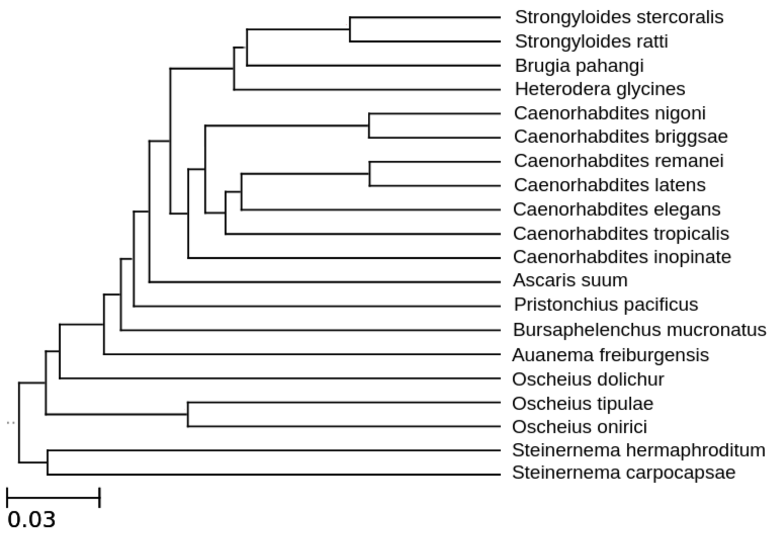

Figure S2. Estimated phylogeny of *Rhabditida* species calculated with `MASH`.

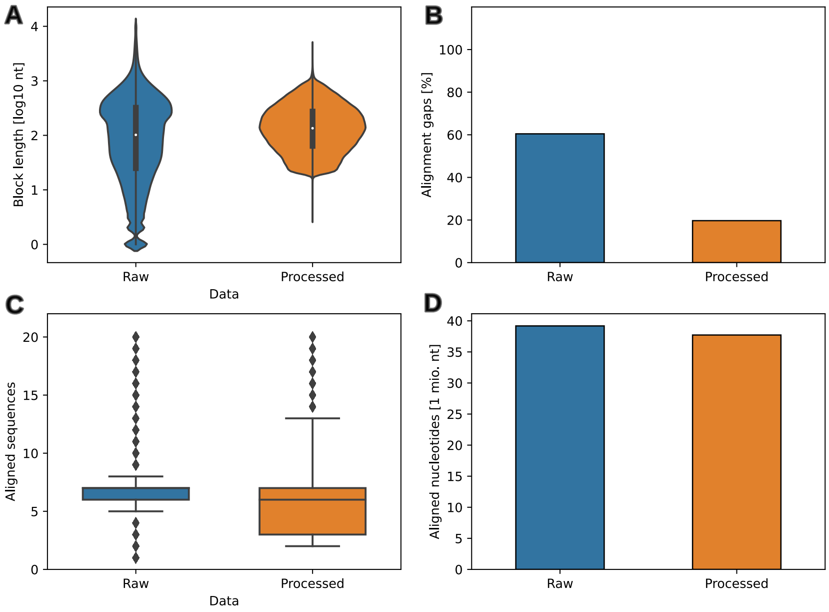

Figure S3. Alignment block statistics before (Raw) and after processing with `MAFtools` (Processed). **A**: Distribution of alignment block lengths in log scale. The variance of block lengths becomes smaller after processing, due to removal of too small and very long but gap-rich alignment blocks. **B**: Total percentages of nucleotides in the *Caenorhabditis elegans* (*C. elegans*) reference genome being only aligned with gaps, the reduction being a result of `MAFtools` rejecting blocks consisting of long, continuous gaps. The remaining 20% are primarily made up of short, internal sections of gap-columns in an existing alignment block. **C**: Number of aligned sequences over all alignment blocks. As sequences are excluded if they only align with short sections of the reference genome to allow for reconciliation with neighboring blocks, the mean number of aligned sequences is slightly lower of the processed data. **D**: Total number of nucleotides of the *C. elegans* genome that are aligned with at least one other nucleotide of another species. With the *C. elegans* genome being ∼100 million nucleotides in length, around 40% of it are covered by the resulting multiple genome alignment. Note that the processing of the genome wide alignment with `MAFtools` is virtually loss-free in terms of genome coverage.

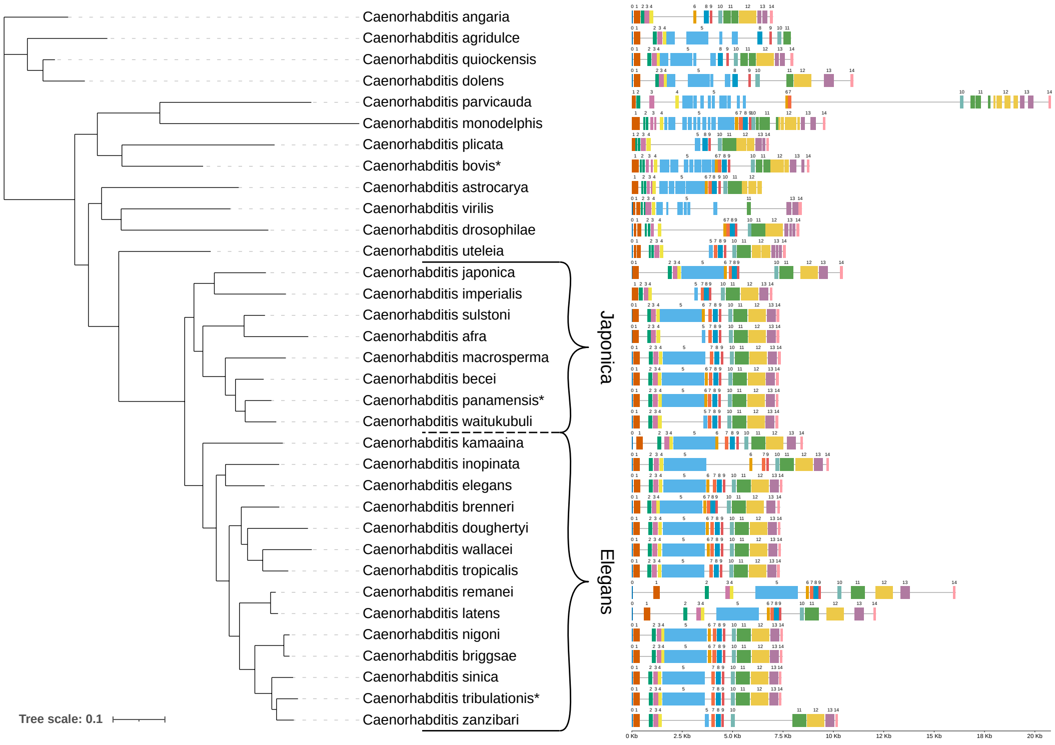

Figure S4. Gene structure of the *nmy-2* gene and it's homologs across *Caenorhabditis* species. Homologs were annotated using the `ExceS-A` tool. Exon lengths are represented by colored rectangles, while intron lengths are marked by the distance between them. Note that the number and exon structure varies between species, complementary sequences (i.e. those corresponding to the same region in the *C. elegans* *nmy-2* gene) are highlighted in the same color. As an example, exon 5 (light blue) is fragmented into many smaller exons in *C. parvicauda* and *C. monodelphis*, among others. The asterisk (*) indicates species without an chromosome level assembly. Gene structure was visualized using `GenomeViz`. The phylogeny was inferred using `Proteinortho` and `IQtree3`, see Figure 4 in the main text.

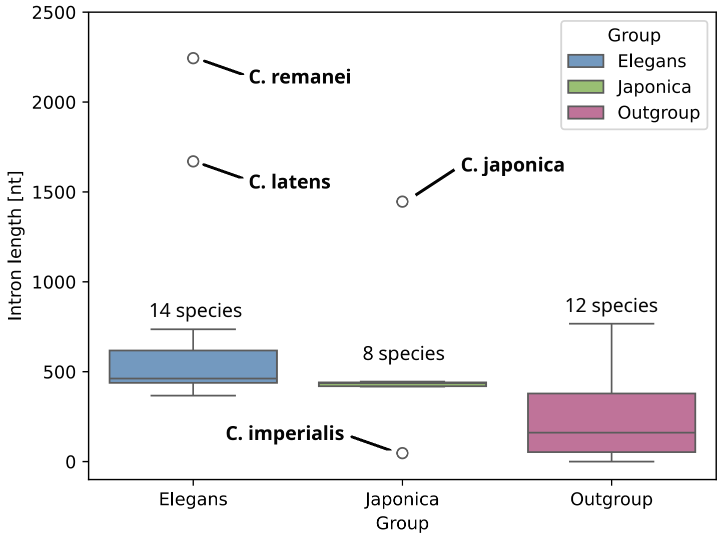

Figure S5. Lengths of the intron 2 equivalent of the *nmy-2* protein in *Caenorhabditis* species. With a few outliers, i.e. *C. remanei*, *C. latens* and *C. japonica*, detected introns of the Elegans and Japonica group show similar lengths of about 500 nucleotides (nts). The distribution of intron lengths for the outgroup is systematically shorter with the extreme of only 42 nt in *C. parvicauda*. There is one unusually short intron present in the Japonica group with *C. imperialis* (47 nt).

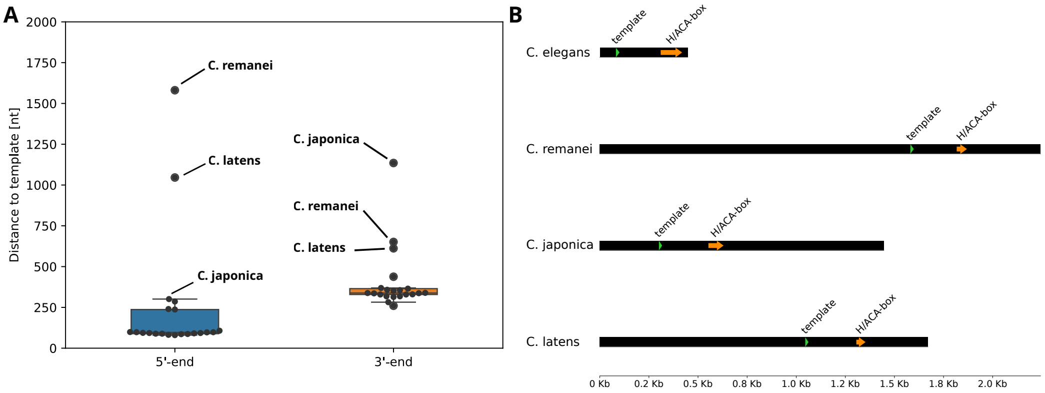

Figure S6. Positioning of identified telomerase RNA template region within the *nmy-2* introns. As it is not known for all introns which part of the premature transcript is clipped during processing, the distance to the termini can be used as a stand-in to estimate telomerase RNA (TR) length and template location. **A**: Offset comparison between the template region and the intron termini. This is very homogeneous between species, with some notable exceptions, namely those that have unusually long intron 2 equivalents (i.e. *C. latens*, *C. remanei* and *C. japonica*). For these cases, it is currently unknown if the TR itself is atypically long or if the processing of transcripts works differently. **B**: Visualization of the template positions in the three extremely long introns in comparison to *C. elegans*. As stated before, the placement of the template (green) is very heterogeneous for the three species. Furthermore the position of the (aligned) H/ACA- box motifs (orange) in all species is illustrated. Note that the position of the `ANANNA` sequence motif in *C. latens* and *C. remanei* is not entirely clear based on the sequence and structure alignment (compare Figure 5) and the annotation above is based on where we would expect it based on the consensus structure model. Arrow length corresponds to the distances between the H and ACA motifs.
